# Monitoring of Hypocalcaemia & Hyperglycemia predictive consequences of Thyroidectomy

**DOI:** 10.1186/1755-7682-7-13

**Published:** 2014-04-01

**Authors:** Syed Wasif Gillani, Diana Laila Rahmatillah, Yelly Oktavia Sari, Mirza R Baig, Syed Azhar Syed Sulaiman

**Affiliations:** 1 School of Pharmacy, Monash University Malaysia, PO box 46150, Bandar Sunway, Selangor, Malaysia; 2 Faculty of Pharmacy, UTA’ 45, Jakarta, Indonesia; 3 Unit of Clinical Pharmacy & Pharmacy Practice, Dubai Pharmacy College, Dubai, United Arab Emirates (UAE; 4 School of Pharmaceutical Sciences, Univerisiti Sains Malaysia (USM), Penang, Malaysia

**Keywords:** Thyroidectomy, Endocrine disorders, Clinical practice, Hyperglycemia, Hypocalcemia

## Abstract

**Background:**

Hyperglycemia and hypocalcaemia have separately been attributed to adverse outcomes in critically ill patients. The study was aim determine whether hyperglycemia and hypocalcaemia together post-operative effect of thyroidectomy and evaluate the gender & age impact on the extend of clinical condition.

**Methods:**

All the patients underwent thyroidectomy in the duration of 1^st^ Jan 2012 till 30^th^ June, 2013 in HPP and HUSM Kelantan, Malaysia. Serum evaluation has been made on 4 consecutive reading with duration of 6 hours. The predictive trend has been established to identify the hypokalemic and hyperglycemic condition. Ethical approvals & Patients’ consent forms have been made prior to conduct this study.

**Results:**

The incidence of hyperglycemia [≥ 150 mg/dl(8.3 mmol/L)] and hypocalcaemia (serum calcium < 8.5 mg/dl (2.2 mmol/L)] were 39.4% and 43.9% respectively. Hyperglycemia and hypocalcaemia associated with age and length of stay, significant association has been found among pre-operative diagnosis as well. The interaction of hyperglycemia and hypocalcaemia did not separate effects on mortality.

**Conclusion:**

As demonstrated, the prevalence of hyperglycemia and hypocalcaemia in post-thyroidectomy patients is considerable high. Also, the linear association pattern has been shown. However, considering the disease severity, the association of hyperglycemia and hypocalcaemia with surgical ward indicators of morbidity could not be verified.

## Introduction

Total thyroidectomy, similar to hemithyroidectomy, is followed by a significant reduction in the plasmatic concentration of PTH [1]. It is associated with a parallel reduction in calcium levels, evident but transitory in a quarter of patients following surgery and permanent in 1% of patients [2]. The frequency of this phenomenon, which can occur during the successive hours after surgery, has recently been the subject of many research programmes, examining the definition of an algorithm that identifies patients with a high risk of postoperative hypocalcaemia [3].

In the modern climate of increasing cost awareness, thyroid surgery has been considered for a 1 day-surgery regime with limiting factors for early discharge being postoperative bleeding (1-2%), bilateral recurrent laryngeal nerve palsy and symptomatic hypocalcaemia [4,5]. Postoperative hypocalcaemia after total thyroidectomy is a serious concern because it is the most frequent complication after thyroid surgery [6-8]. It is usually evident in the first 24 hours.

A reliable method that could accurately identify patients who are at high risk for hypocalcaemia may assist in the selection of patients suitable for early discharge [3]. The aim of the present work is to evaluate the risk of hypocalcaemia following thyroid surgery and to determine whether early serial postoperative serum calcium levels after total thyroidectomy can be used to develop an algorithm identifying patients who are unlikely to develop significant hypocalcaemia and can be safely discharged within 24 hours after surgery.

There is an open debate in scientific literature regarding the use of early parathyroid hormone (PTH) levels as a predictor of significant hypocalcaemia [9]. Similarly in the most recent studies, hyperglycemia and hypocalcaemia have separately been attributed to adverse outcomes including higher mortality and longer length of hospitalization in critically ill patients [4-11]. However, such studies suffer from some shortcomings. First, most of them have been conducted on critically ill adult patients and secondly, most authors have evaluating their interactions with mortality and length of stay (LOS).

The objective of this study was to determine whether hyperglycemia and hypocalcaemia together post-operative effect of thyroidectomy and evaluate the gender & age impact on the extend of clinical condition.

## Material and methodology

The settings for the study were Hospital pulau Pinang (HPP) and Hospital University Sains Malaysia (HUSM) Kelantan, both are tertiary care hospitals. All the patients’ undergone surgery of thyroidectomy was eligible to be included in the study. Duration of the study was from 1^st^ Jan 2012 till 30^th^ June 2013. Ethical clearance has been made according from the Centre of Research Committee (CRC) and Ministry of Health (NIH-MREC). Prior to study patients’ consent forms have been obtained.

**Figure 1:**
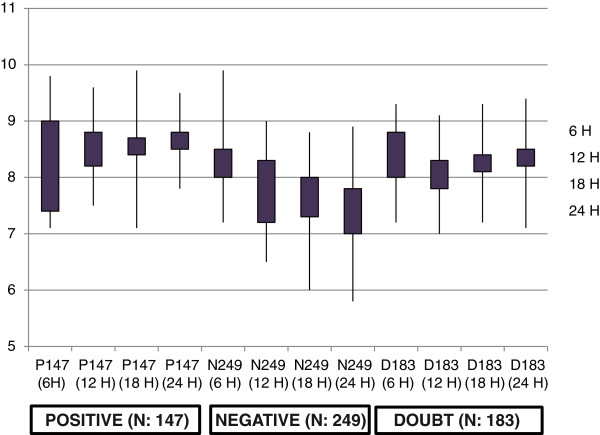
Distribution of calcium levels according to dose and trend.

The independent variables were initial blood sugar (RBS) and serum calcium (Ca) within the first 6 hours of surgery. Serum calcium and BS (RBS) measurements at 6, 12, 18 and 24 hours postoperatively were evaluated for each patient. A calcium level of 8.3 mg/dL was considered as a threshold value of hypocalcaemia, defining the following: a “negative trend” when the straight halfway line of the 3 values had a decreasing tendency, a “positive trend” when the straight halfway line of the 3 values had an increasing rise and a “doubt trend” when there was a decrease arriving near but over the threshold value of hypocalcaemia (Figure 1). Hypocalcaemia was defined as experiencing signs or symptoms of hypocalcaemia perioral and digital paresthesias (Chvostek’s sign and Trousseau’s sign) and/or having a serum calcium level that was lower than8.2 mg/dL. Patients were placed in a treatment algorithm based on the level of serum calcium and on the signs or symptoms of hypocalcaemia (calcium gluconate1-2 g/day and calcitrol 50–100 μg/day [10,11]. Similarly patients were classified into two groups based on their blood sugar levels. Group1 (Controlled) with RBS < 150 mg/dl (8.3 mmol/L) and group 2 (hyperglycemic) with RBS ≥ 150 mg/dl (8.3 mmol/l) [12].

According to the change in the serum calcium level among the 4 measurements, a variant analysis was performed, using p-values in order to identify the possibility of reducing the number of measurements necessary to evaluate the risk of hypocalcaemia. The data were compared, using student’s T-test for continuous, normal distributed variables, and the Mann–Whitney test for non-normally distributed variables. Categorical variables were compared, using the chi-square or Fisher Exact test. Logistic regression analysis was used to analyze the interaction of the two-variables. *P* value less than 0.05 was considered as significant. Statistical analysis was performed using SPSS software (version 20® Chicago, IL, USA).

## Results and findings

The study was comprised of 579 patients who had undergone surgery for various thyroid diseases (goiter, cancer, etc.). The mean age of the patients was 47 (range 55–79), 388 of the patients were females and 191 were male (Table 1).

**Table 1:** Distribution of patients’ undergone surgery

	**N (%)**
**Age** (Mean ± SD)	49 ± 5.769
Range 35–79 years	
**Sex**	
Male	191 (33.0)
Female	388 (670.)
**Clinical diagnosis**	
Cancer	210 (36.3)
Adenoma	171 (29.5)
Inflammatory	62 (10.7)
Goiter	136 (23.5)
**Post-Operative**	
Hypocalcaemia	254 (43.9)
Hyperglycemia	228 (39.4)

Then Mean ± SD of LOS was 4.68 ± 4.93 days which ranged from 1 to 20 days. The initial RBS ranged from 15 mg/dL (0.87 mmol/L) to 640 mg/dL (37.27 mmol/L) with mean ± SD of 144.63 ± 85.94 mg/dL. The incidence rate of hyperglycemia [i.e. RBS ≥ 150 mg/dL (8.3 mmol/L)] was 39.4%. During inpatient stay in surgical ward, 87.5% of the patients had their total serum calcium (Ca) checked with a range of 5.4-11.9 mg/dL (1.33-2.94 mmol/L) [8.72 ± 1.12 mg/dL (mean ± SD)]. The incidence rate of hypocalcaemia [i.e. Ca < 8.5 mg/dL (2.12 mmol/L)] was 43.9%. Among the patients, Ca values significantly effect on the LOS (p = 0.020 but no effect 0.78 with RBS) (Table 2). While the mean age has a significant effect with RBS ≥ 150 mg/dl.

**Table 2:** Blood sugar and Calcium levels among study population

	**Calcium level**		**P-value**	**Random blood sugar**		**P-value**
	**Normal**	**Hypo**		**Controlled**	**Hyper**	
**Age** (Mean ± SD)	46 ± 4.514	47 ± 4.412	0.32*	46 ± 4.141	50 ± 4.822	0.021*
**Sex**						
Male	105 (32.3)	86 (33.9)	0.89	119 (33.9)	72 (31.6)	
Female	220 (67.7)	168 (66.1)		232 (66.1)	156 (68.4)	0.77
**Clinical diagnosis**						
Cancer	30 (9.2)	180 (70.9)		49 (14.0)	161 (70.6)	
Adenoma	137 (42.2)	34 (13.4)	0.01	149 (42.4)	22 (9.6)	
Inflammatory	35 (10.8)	27 (10.6)		42 (12.0)	20 (8.8)	0.001
Goiter	123 (37.8)	13 (5.1)		111 (31.6)	25 (11.0)	
**LOS** (Mean ± SD)	4.55 ± 4.14	6.82 ± 5.87	0.020*	4.38 ± 5.05	4.98 ± 5.72	0.78*

LOS, Length of Stay; *P vale from Student T-test, Chi-square for rest.

Hypocalcaemia was associated with 3.9 fold increase in the risk of hyperglycemia (95% confidence interval 1.245-5.742, p = 0.001) (Table 3).

**Table 3:** Association and covariance of hypocalcaemia with hyperglycemia

**Blood sugar**	**Normocalcemia**	**Hypocalcaemia**	**P-Value**	**Total N (%)**
	**< 8.5 mg/dl (2.2 mmol/l)**	**≥ 8.5 mg/dl (2.2 mmol/l)**		
**Controlled**	315 (96.6)	36 (14.2)		351 (60.6)
**< 150 mg/dl (8.3 mmol/l)**			0.000*	
**Hyperglycemia**	10 (3.1)	218 (85.8)		228 (39.4)
**≥ 150 mg/dl (8.3 mmol/l)**				
**RR (95% CI)**	3.9 (1.24 – 5.742 )		0.001*	
**Odd ratio**	190.75			
**95% CI**	92.7007 – 392.5058		0.000*	
**Z-Statistics**	14.263			
**Total**	325 (56.1)	254 (43.9)		579 (100.0)

* < 0.05.

Thyroid pathologies, gender did not show any significant correlation to the development of significant post-operative hypocalcaemia and hyperglycemia. From 579 patients studied, 147 (25.4%) showed a positive trend, only 15 of these (10.3%) successively developed hypocalcaemia; from 249 patients with a negative trend 212 (85.2%) developed hypocalcaemia and with a doubt trend only 27 (14.7%) of 183 patients (31.6%). As expected, the incidence of hypocalcaemia was moderate for patients with a doubt trend and maximal in patients with a negative trend. On the other hand, the findings of a negative trend calcium level was a absolutely predictive for hypocalcaemia; in fact it developed in 212 patients (85.2%) (Figure 1).

A positive slope predicted hypocalcaemia 10.3% and a negative slope predicted 85.2% of the time, 10% probability of remaining within normal calcium levels (Table 4). Patients were treated with oral calcium in the case of hypocalcaemia. At three months after surgery 97.08% of patients had normal serum calcium levels. This demonstrated that by solely relying on a positive or negative rise trend, there was still a significant risk of the development of hypocalcaemia. Similar pattern has been found with hyperglycemic predicting value. The findings suggested the parallel linear association between hypocalcaemia and hyperglycemia. We did not find any statistically significant differences in the plasma calcium level and RBS between the samples taken at 18 and 24 hours after surgery (Table 4).

**Table 4:** Distribution of patients according to trend and post-operative condition

**Trend**	**N (%)**	**Normocalcemia**	**Hypocalcemia**	**Hyperglycemia**	**Control**	**N (%)**	**Trend**
**Positive**	**147 (25.4)**	**132 (89.7)**	**15 (10.3)**	**180 (62.3)**	**109 (37.7)**	**289 (49.9)**	**Positive**
	**249 (43.0)**					**67 (11.6)**	
**Negative**		**37 (14.8)**	**212 (85.2)**	**38 (56.7)**	**29 (43.3)**		**Negative**
	**183 (31.6)**					**223 (38.5)**	
**Doubt**		**156 (85.3)**	**27 (14.7)**	**10 (4.5)**	**213 (95.5)**		**Doubt**
**Total**	**579 (100)**	**325 (56.1)**	**254 (43.8)**	**351 (60.6)**	**228 (39.4)**	**579 (100)**	**Total**

## Discussion

Improvement in surgical technique has led to a relevant decrease in severe postoperative complications after thyroid surgery and surgeons are considering whether one day hospital would be feasible after total thyroidectomy. In fact severe hypocalcaemia continues to represent a limiting factor for such a short stay in hospital.

The present study demonstrated that hyperglycemia and hypocalcaemia are the post-operative clinical consequences of thyroidectomy. The effect substantially increase the length of stay, also predicting the negative trend among these independent variables for initial assessment would be beneficial. To the best of our knowledge, there has been no reported study on this issue. Meanwhile, the results of the present study revealed that hyperglycemia and hypocalcaemia were significantly more prevalent among the pre-operative diagnosis. These findings have revealed among critically ill surgical patients [13-15]. It has also been demonstrated that even moderate degree hyperglycemia [RBS > 110 mg/dl (6.11 mmol/L)] was associated with mortality in ICU [16]. Hirshberg and her colleagues also showed that hyperglycemia and glucose variability are associated with increased prevalence of nosocomial infection in critically ill patients [17].

Although Klein and his colleagues in a recent study claimed that hyperglycemia was not independently associated with increased complications and LOS [18], our study revealed significant association with hypocalcaemia and LOS independent to hyperglycemia. It seems that it is somehow the reflection of the disease severity and not simple the deleterious effect of hyperglycemia as it was asserted by Srinivasan and his Co-workers [16].

Moreover, like previous studies [16,19], the present study investigated the traditional approach of permissive hyperglycemia with blood glucose just below the renal threshold (180–200 mg/dl) (10–11.11 mmol/L) in critically ill patients. It seems that tight glucose control might have favorable effects on the outcomes of patients admitted to intensive care units [18], although this approach has been recently y questioned by Klein and his colleagues in their large retrospective study [17].

The incidence of hypocalcaemia after total thyroidectomy – transient in the majority of cases – in literature oscillates between extremely large limits (from 11.2% to 35%) [6-8]. Regarding our investigation, we have observed a postoperative hypocalcaemia in 43.9% of the cases.

There is no existing scoring method allowing the identification of patients who will not develop severe hypocalcaemia. We used early serum calcium levels after total thyroidectomy to identify patients with a risk of developing significant hypocalcaemia and allowing an early discharge. The positive rise of the calcium level after total thyroidectomy is a reliable method in 96.2% of cases allowing the patient to be discharged with a risk of hypocalcaemia of only 3.8%. Otherwise, the doubt trend gives a 16.9% margin of risk, making it necessary to conduct further hematic measurements in the consequent hours.

In the case of negative trend, the risk of hypocalcaemia is 85.2%. In this case, the decreasing tendency of serum calcium level remains an imperfect method but anyway the hospital discharge is delayed. Regarding the serum calcium measurements used to evaluate the trend, we found no statistically significant calcium level at 18 hours after surgery. Intra and postoperative intact parathyroid hormones has been embraced with enthusiasm by many surgeons as a means to detect patients with the highest risk of severe hypocalcaemia, but its major limitation for wider clinical use is the cost factor.

## Conclusion

As demonstrated, the prevalence of hyperglycemia and hypocalcaemia in post-thyroidectomy patients is considerable high. Also, the linear association pattern has been shown. However, considering the disease severity, the association of hyperglycemia and hypocalcaemia with surgical ward indicators of morbidity could not be verified. In addition, the interaction of both disturbances did not have any synergistic effect on mortality or morbidity. It seems that more prospective, randomized multi-center trials are needed to consolidate the findings and help make more proper judgment.

## Competing interest

The authors declare that they have no competing interests.

## Authors’ contribution

All authors read and approved the final manuscript. **SWG (**wasifgillani@gmail.com**):** principle investigator. **DLR (**dianalailaramatillah@gmail.com**):** data analysis and variable identification. **YOS (**yosari_15@yahoo.com**):** data collection and arrangement. **MRB (**pharma_baig@rediffmail.com**):** data interpretation. **SASS (**sazhar@usm.my**):** Project supervisor and content evaluation.
